# “We may conclude that:” a corpus-based study of stance-taking in conclusion sections of RAs across cultures and disciplines

**DOI:** 10.3389/fpsyg.2023.1175144

**Published:** 2023-05-05

**Authors:** Liming Deng, Ping He

**Affiliations:** Foreign Language Research Institute, College of Foreign Languages and Literature, Wuhan University, Wuhan, China

**Keywords:** research articles (RAs), stance, cross-cultural variation, conclusion section, disciplinary differences

## Abstract

Research article conclusions form an important sub-genre in the academic community. This study aims to compare the use of stance markers in English and Chinese research article conclusions and investigate how stance markers may vary in soft and hard sciences. Based on Hyland's stance model, an analysis of stance markers over 20 years was made in two corpora, which were compiled with 180 research article conclusions in each language from four disciplines. It was found that English writers and soft science writers tended to make statements more tentatively by hedges and craft their persona more explicitly through self-mentions. However, Chinese writers and hard science writers made their claims with more certainty by boosters and showed their affective attitude more frequently through attitude markers. The results reveal how writers from different cultural backgrounds construct their stances and also unveil the disciplinary differences involved in stance-taking. It is hoped that this corpus study will inspire future research on stance-taking in the conclusion section and also help cultivate writers' genre awareness.

## 1. Introduction

Stance has aroused considerable attention in academic writing research, particularly in the research articles (hereafter RAs) genre, in recent years (Hyland, 2005; Chen, 2020). This is due to the role it plays in competent academic writing (Hyland, 2012) and the incrementally acknowledged belief that academic writing is not purely “objective and impersonal” (Hu and Cao, 2011; Liardét and Black, 2019) but expresses writers' stance (Vold, 2006; Loi et al., 2016). Stance, being the indispensable part of interpersonal resources to show writers' textual voice, is defined as “attitudes, feelings, judgments, or commitment concerning the propositional content of a message” (Biber and Finegan, 1989, p. 93). Hyland further categorizes stance as “writer-oriented features of interaction” (Hyland, 2005, p. 178) or the “writer's rhetorically expressed attitude to the proposition in a text” (Hyland, 2012, p. 134). Therefore, not only disciplinary knowledge and claims but also writers' attitudes are normally conveyed by stance markers. Accordingly, it is important for academic writers, especially for non-native and novice scholars who encounter difficulty in presenting claims and authorial stances properly (Crismore et al., 1993; Abdollahzadeh, 2011), to grasp an in-depth understanding of stance-taking in academic writing.

While previous studies have provided noteworthy evidence on the deployment of stance markers across English and Chinese RAs, there are some lacunae to fill in. First, some studies (e.g., Mu et al., 2015; Li and Xu, 2020) only focused on one discipline with limited data. Second and most important, previous studies scarcely delved into the systematic use of stance markers in the specific conclusion section. Hence, it is important to explore how stance markers are employed across different languages/cultures, not only for English and other Indo-European languages but also for Chinese, “a dominant Sino-Tibetan language and culture, which is particularly worth more attention” (Li and Xu, 2020, p. 48). Furthermore, given the importance of the conclusion section in RAs, which serves as the last chance to highlight the importance of results and persuade readers (Yang and Allison, 2003), stance markers might play a different role within the section from the other sections in RAs.

Therefore, the current study analyzes the use of stance markers across Chinese and English RAs in the conclusion section of four disciplines (i.e., soft sciences with applied linguistics and sociology and hard sciences with mechanical engineering and biology) based on two self-built corpora. It aims to help non-native and novice research article writers know how academic writing is influenced by different factors and better present themselves in their writing within the boundary of academic convention.

## 2. Literature review

### 2.1. Stance-taking in academic writing

Several studies have examined how writers show their stance over the last decade (e.g., Hyland, 2005, 2012; Alghazo et al., 2021; Liu et al., 2022), indicating cultural and disciplinary differences due to the tendency of writers to construct and negotiate social relations in their writing (Hyland and Tse, 2004). Some scholars discussed the use of stance markers in student writing, such as essays and reports (Crosthwaite and Jiang, 2017), graduate theses (Charles, 2006), master theses (McCambridge, 2019), and dissertation writing (Liu et al., 2022). These studies indicate that different linguistic choices of stance markers are used to show writers' opinions and attitudes, which can be attributed to different English proficiency levels.

Other scholars focused on specific linguistic choices for stance-taking in RAs, such as nouns (Charles, 2003; Jiang, 2017), verbs (Vold, 2006), adverbials (Biber and Finegan, 1989), conditional clauses (Warchał, 2010), reporting clauses (Charles, 2006), and meta-discourse features (Yang, 2013; Li and Xu, 2020), which help us to get a grip on how stance markers are used in RAs across cultures and disciplines. It can be stated that stance markers play a significant part in RA writing. For instance, hedges can show writers' prudence in their statements, based on plausible reasons, and involve readers by opening a discursive space (Hyland, 2005). Boosters facilitate solidarity and assurance pertaining to the results (Hu and Cao, 2011). Attitude markers are conducive to the display of the importance of, agreement on, and obligation to the statements (Mur-Dueñas, 2011; Hu and Cao, 2015). Finally, self-mentions are essential to identity construction (Walková, 2019; Chen, 2020) and authorial presence (Hyland and Jiang, 2019). Suffice to say, RA writing, as a disciplinary and professional practice, not only displays the propositional content but constructs the epistemological stance of the writer. By taking stance strategies, writers could better negotiate their stance in RAs while maintaining the discourse in a scrutinized and coherent way that is more acceptable to the discourse community.

### 2.2. Research article conclusion sections

Different RA sections serve distinct communicative purposes (Swales, 1990). The conclusion section, serving as the closing section and last chance to present results and highlight the significance, and thus as an important sub-section in RAs (for example, 26 out of 31 of the RAs published in *English for Specific Purposes* in 2015 had separate conclusion sections), plays a big part in achieving the communicative purposes of summarizing and emphasizing the results, highlighting the contribution and providing future directions (Yang and Allison, 2003; Sheldon, 2019). However, it has received less attention in comparison with the *abstract* (Alghazo et al., 2021), *introductions* (Loi and Lim, 2013), and *discussions* (Li and Xu, 2020). This may be due to the classification of the *conclusion* as a *discussion* following Swales (1990)' IMRD model. However, Peacock (2002) identified the communicative moves of discussion and conclusion sections in seven disciplines and proposed that the conclusion section had its distinctive communicative purposes of *summarizing and highlighting the study* compared with the discussion section of *evaluating the results*. Following his study, Yang and Allison (2003) found that 13 out of 20 RAs in their corpus had separate conclusion sections and illuminated that the conclusion section should be treated as an independent section but not as an adherence to the *discussion*. Hence, they established a three-move model of RA *conclusions* through genre analysis of 20 RAs in applied linguistics.

Some scholars have examined the use of stance markers in *conclusions*. Chen and Zhang (2017) have found that Anglophone academic English writers used more hedges in *conclusions* of applied linguistic RAs with different linguistic expressions than their Chinese counterparts. Abdollahzadeh (2011) conducted a systematic comparative study of interpersonal markers in 60 *conclusions* of applied linguistics RAs written in English by Anglo-American and Iranian academic writers. He reported that both groups showed a tendency for hedges, while differences existed in the higher use of emphatics and attitude markers by Anglo-American authors. Later, Mu et al. (2015) identified that English academic writers tended to use more interactional markers than Chinese writers, with a significant difference in hedges, boosters, attitude markers, and self-mentions from the overall generic organization in applied linguistics RAs.

Previous research from an intercultural perspective has offered an insight into the exploitation of stance markers in RA *conclusions*. In addition, it is widely accepted that academic writers engage themselves in their writing ascribed to the cultural and disciplinary communities (Dahl, 2004; Vold, 2006; Loi et al., 2016; Ädel, 2022). To date, little research has focused on the use of stance markers in RA *conclusions* from the cross-cultural/linguistic and cross-disciplinary perspectives. Therefore, we attempt to explore how stance markers are employed in RA *conclusions* by English and Chinese writers within different disciplines' backgrounds. Specifically, we attempt to address the following research questions:

1. What are the similarities and differences in the use of stance markers between the Chinese and English RA conclusion sections?
2. How do Chinese and English writers employ stance makers in soft and hard sciences RA conclusion sections?

## 3. Corpus and procedures

### 3.1. Corpora

To address the research questions, RA *conclusion*s were extracted from four different disciplines, applied linguistics and sociology for soft sciences and biology and mechanical engineering for hard sciences. English and Chinese texts from the four disciplines were selected to compile a corpus in each language. The four specific disciplines were chosen because they were established representatives of their respective disciplinary groupings that were often selected in previous studies (Hyland, 2005; Hyland and Jiang, 2016). On the other hand, this study adopted a more stringent way of categorization, with sections under the heading “Discussions and Conclusion” being excluded. All the “Conclusion” sections (marked as a separate section) in this corpus were (i) placed immediately after the Discussion section; (ii) contained an overall summary or conclusion with the implication and/or limitations; and (iii) followed by the References and/or Acknowledgments and/or Supplementary material Appendix sections. Furthermore, to make the two groups of texts comparable, 24 journals were selected from the Web of Science and China National Knowledge Infrastructure databases. The sample journals were selected with the consideration of the impact factor, the representative of the discipline, the suggestion from experts in each discipline, and the occurrence in previous studies (Hyland and Tse, 2004; Hyland, 2005; Hyland and Jiang, 2016). Each discipline included three English journals and three Chinese journals. Altogether, 360 RA conclusions (i.e., 15 articles from each of six leading journals in four disciplines) were selected randomly from the years 1995, 2005, and 2015 (to be more persuasive by covering a long period), with 180 in each language, respectively. Finally, two sub-corpora were comprised: (1) the English RA corpus (i.e., English conclusions written by English speakers published in international English journals) and (2) the Chinese RA corpus (i.e., Chinese conclusions written by Chinese speakers published in Chinese journals; see Table 1).

**Table 1:** Description of the corpus.

		**No. of conclusion**	**English sub-corpus**		**Chinese sub-corpus**			**Total**
			**No. of English words**	**Mean**	**No. of Chinese characters**	**Mean**	**Total**	
Soft	AL	90	21,597	243.97	15,370	170.78	36,967	87,838
	SOC	90	28,028	311.42	22,483	249.81	50,871	
Hard	ME	90	11,220	124.67	9,562	106.24	20,782	47,556
	BIO	90	10,586	117.62	16,188	179.87	26,774	
Total		360	71,431		63,963			135,394

AL, applied linguistics; SOC, sociology; ME, mechanical engineering; BIO, biology.

### 3.2. Analytical framework

To identify stance markers in our corpus, we adopted Hyland's (2005) stance model for two concerns. First, it aims to interpret how writers construct their stance in the discourse community with a top-down, comprehensive, integrated, and accurate taxonomy, which is appropriate for our study. Second, this model has been widely used in recent studies (e.g., Abdollahzadeh, 2011; Mur-Dueñas, 2011; Hyland and Jiang, 2016), including the comparison between Chinese and English (e.g., Mu et al., 2015). Table 2 presents the main types of stance markers illustrated with examples from our corpus.

**Table 2:** Taxonomy of stance in Chinese and English (Hyland, 2005).

**Types**	**Functions**	**English examples**	**Chinese examples**
Hedges	Withhold writer's full commitment to statements	May, might, could, would	*Keneng* (may), *yidingchengdu* (to some extent)
Boosters	Stress force or writer's certainty	Can, will, must, in fact	*Bixu* (must), *zhengming* (prove), *keyi* (can)
Attitude markers	Express writer's attitude including significance, obligation to proposition	Should, agree, surprisingly, remarkable	*Yinggai* (should), *xiwang* (hope), *zhongyaode* (important), *zhide* (deserve)
Self-mentions	Refer to author(s)	I, we, our	*Women* (our), *bizhe* (the author)

As for the above taxonomy of stance, it can be seen that four dimensions are involved. The first is concerned with hedges. A hedge is a device to mitigate the writer's decision and withhold complete commitment to a proposition (Hyland, 2005) that is realized by varieties of linguistic choices, such as modal verbs (may, could), adjectives and adverbs (possible, perhaps), nouns (possibility, probability), and phrases (be likely to, seems to). Thus, hedges show writers' prudence or detachment in their statements and also involve readers by opening a discursive space (Vassileva, 2001) to construct their dual stance or identity of a *humble servant* (Hyland, 2001) in the discourse community and a *producer* of new knowledge in social interactions with community members.

The second category of stance markers is about boosters. Boosters, contrary to hedges, increase the writer's certainty and boost the claims concerning the propositional content. Boosters in RAs are achieved by modal verbs (can, must), verbs (demonstrate, prove), adjectives and adverbs (clear, certain/ly), and phrases (no doubt, in fact). The appropriate use of boosters can consolidate the solidarity with readers and assurance of the statements (Hu and Cao, 2015).

The third is related to attitude markers. Attitude markers generally show writers' affective attitudes and evaluations of propositions captured by modal verbs (should, need), verbs (believe, hope), adjectives and adverbs (important, fortunately), and phrases (in particular). These devices explicitly embrace writers' affective attitudes or evaluations of materials such as “surprise, agreement, importance, frustration, and so on” (Hyland, 2005, p. 180). Therefore, attitude markers help writers persuade readers by revealing the socially recognized value system and foregrounding shared attitudes in a discourse community (Hu and Cao, 2015).

The last is related to self-mentions. Self-mentions present propositional, affective, and interpersonal information (Hyland, 2005). They explicitly allow writers to construct their stance through the use of first-person pronouns (I, we), possessive determiners (our, my), and the less subjective phrase (the author). As highlighted by scholars (Kuo, 1999; Hyland, 2001; Walková, 2019), self-mentions can be conducive not only to the discourse organization but to the emphasis of writers' contributions and feelings.

The abovementioned four dimensions of stance-taking indicate that writers do not just merely present ideas in ways that are comprehensible and persuasive to a target audience but convey their personality, reliability, and relationship to a proposition or statement as well.

### 3.3. Procedure and reliability

The research was conducted with the following procedures:

Stage 1: We selected RAs of each discipline from the years 1995, 2005, and 2015, respectively. However, we did encounter a problem in that some journals did not cover five original RAs with a separate conclusion section in the year 1995. Thus, we had to supplement these with others from the published RAs in 1996.
Stage 2: We filtered the above-selected articles by excluding those without a separate conclusion section (e.g., titled as *discussion and conclusion*). Then, we transformed the pdf or caj into a text version for the convenience of coding.
Stage 3: The text was annotated manually through careful reading. First, stance markers were identified and classified into four categories. Next, we tried to find the Chinese stance markers corresponding with the English stance markers and then identified and classified them in the situated context to keep consistency with the above categories.
Stage 4: To ensure the reliability of annotation, half of the data were first annotated by the authors according to Hyland's (2005) framework. A coding reliability coefficient of 0.88 was reached, indicating a good level of inter-coder reliability. Then, we discussed the inconsistencies in the coding process and revised the coding scheme. After that, one of the authors completed the coding process of all these research articles.

Finally, chi-square analyses were carried out on the stance markers' sub-corpora to observe if there were any statistically significant differences in the frequency of stance markers across cultures and disciplines.

## 4. Results

To answer the research questions, both quantitative statistical analysis and qualitative textual analysis were conducted. The statistical analysis focused on the occurrence and frequency of the stance markers in the corpora. The textual analysis was performed to identify the functions and prominent patterns of the stance markers.

According to the result of the chi-square test (χ^2^ = 142.185, df = 3, *p* < 0.05), there is a statistically significant difference between the frequencies of stance markers in the English sub-corpus and Chinese sub-corpus, as displayed in Table 3. Overall, stance marker features are overwhelmingly more salient in English than in Chinese. Similarly, the results between the soft and hard sciences both in the English sub-corpus and Chinese sub-corpus are also significant (see Table 4). The overall occurrence of stance markers in soft sciences far surpasses that in hard sciences. The detailed analysis is presented below.

**Table 3:** Presentation of the chi-square test of independence by language.

	**Chinese sub-corpus**		**English sub-corpus**		**χ^2^**	***p***
	**Raw number, %**	**Per 10,000 characters**	**Raw number, %**	**Per 1,000 words**		
Hedges	57 (11.90%)	8.9	**441 (24.80%)**	61.7	142.185	0.000
Boosters	**163 (34.03%)**	25.5	483 (27.17%)	67.6		
Attitude markers	**194 (40.50%)**	30.3	340 (19.12%)	47.6		
Self-mentions	65 (13.57%)	10.2	**514 (28.91%)**	72.0		
Total	479 (100%)	74.9	**1,778** (100%)	248.9		

For those bold numbers, the number is greater than the counterpart.

**Table 4:** Presentation of the chi-square test of independence by discipline.

		**Hedges**	**Boosters**	**Attitude markers**	**Self-mention**	**Total**	**χ^2^**	***p***
Chinese sub-corpus	Soft	**41 (12.65%)**	105 (32.41%)	117 (36.11%)	**61 (18.83%)**	**324 (67.64%)**	26.410	0.000
	Hard	16 (10.32%)	**58 (37.42%)**	**77 (49.68%)**	4 (2.58%)	155 (32.36%)		
English sub-corpus	Soft	**318 (25.77%)**	296 (23.99%)	218 (17.67%)	**402 (32.57%)**	**1,234 (69.4%)**	39.763	0.000
	Hard	123 (22.61%)	**187 (34.38%)**	**122 (22.43%)**	112 (20.58%)	544 (30.6%)		

For those bold numbers, the number is greater than the counterpart.

### 4.1. The use of stance markers across the Chinese and English RA conclusion sections

#### 4.1.1. The statistical analysis of similarities and differences

Initially, the similarities in the occurrence of the stance markers will be displayed. As shown in Table 3, the raw number of boosters is the second most common stance marker both in the English sub-corpus and Chinese sub-corpus. However, the occurrence of stance markers in the English sub-corpus markedly differs from that in the Chinese sub-corpus. First, stance markers are overwhelmingly more common in English RAs than in Chinese ones, as the overall frequency of English stance markers is 248.9 per 10,000 words, while it is only 74.9 per 10,000 characters in Chinese (*p* < 0.05). Second, the occurrence of the four sub-categories in the English sub-corpus is more varied (e.g., the standard deviation is 72.02) than that in the Chinese sub-corpus (e.g., the standard deviation is 53.45). Third, it is found that English RAs tend to use more hedges (24.8 > 11.9%) and self-mentions (28.91 > 13.57%), while Chinese RAs tend to use more boosters (34.03 > 27.17%) and attitude markers (40.5 > 19.12%).

#### 4.1.2. The textual exhibition of stance markers functions

Based on the textual analysis, we can observe that arguments are hedged simply to show the uncertainty and ambiguity and gain acceptance from the postulated readers in both the English and Chinese sub-corpus. However, English writers used more hedges to make an inference or to mitigate the results. For example:

(1) Perhaps *this is a result of the trend of globalization in the academic world* (E-AL-ESP-09).

In the Chinese sub-corpus, hedges were mainly used to show politeness, humility, and deference to avoid face-threatening acts from the readers or authorities. For instance:

*(2) [At present*, we may not *dare to make haste judgments personally.]*^1^ *(C-SOC-YS-12)*.

Boosters, however, were used to show certainty, commitment, and confidence in both English and Chinese RAs. Meanwhile, a difference does exist. In the English sub-corpus, writers employed boosters to highlight the significance and contribution of the research results. However, Chinese writers employed boosters to stress the common knowledge concerning the findings. Look at examples below:

*(3) The proposed measures* can also be *employed in first language acquisition research and in comparative linguistics (E-AL-SLR-14)*.
*(4) [The integration of [sic]electric vehicle into [sic]power system* can *affect the normal operation of [sic]power grid with* no doubt*] (C-ME-JMEE-15)*.

As it is noted that “hedges and boosters are the two sides of the same coin” (Hu and Cao, 2011, p. 2,796), it is an important competence for writers to bind these two devices in the same writing. It has been revealed that English writers have a better awareness of joining the two sides together to keep the balance of qualifying the results with uncertainty and strengthening the commitment with tentativeness, which can both underscore the arguments and leave room for the reader's negotiation to consolidate the solidarity with potential community members. Also, by the use of *inclusive we*, the author can better present their persona by moderating the responsibility. For example:

*(5) While these approaches* are certainly logical and useful, we might *raise some of the same questions about their efficacy that* we *ask about the effectiveness of teaching grammar, usage, and style in a lecture or discussion format (E-AL-JOSLW-02)*.

Attitude markers are utilized for showing the importance of the findings, agreement/disagreement with previous studies, obligation and expectation for upcoming researchers, and personal emotions of surprise and frustration. Although the difference in raw number is the least among the four sub-categories, personal emotions were identified more in English than in Chinese. This can be seen in Example (6), where the writer further highlighted the affective feeling by supplementing *particularly* after *surprising*.

*(6) This is* quite surprising *considering its significance to human health worldwide*, particularly *its high potential for causing devastating epidemics (E-BIO-JOTB-08)*.

Nevertheless, Chinese writers emphasized providing guidance for future studies and policymakers. They would give suggestions to policymakers to solve real-world problems based on their findings [see Example (7)].

*(7) [Under the current circumstances*, the government needs to *consider how to improve the household registration system and also* needs to *further straighten out the land and property system] (C-SOC-YS-09)*.

Next, though *we* and *our* were shown in both the English and Chinese sub-corpus to show writers' authorial stance, Chinese writers deployed the more *inclusive we* to show the belonging of the community by hiding the individual and showing their modesty. For instance:

*(8) [Thus, in the teaching of newspaper reading*, we should *emphasize the importance of the environment and cultural background] (C-AL-FLW-04)*.

Also, the use of *we* instead of *I* in Chinese could eschew the responsibility even though there is only one writer for a single RA. Furthermore, *the author* was used to distance the writers from an authority that might mitigate the responsibility of the writer as an individual. Look at the following examples:

*(9) [In this paper*, we *illustrate the code mixing discourse as a marked form of expression] (C-AL-FLW-01)*.
*(10) [*The author *applied the self-learning control theory into the active vibration control of a rotor system with periodic excitation] (C-ME-CME-04)*.

In contrast, *I* was found to be quite common in English RAs to show the writer's responsibility and personal commitment by sometimes combining with attitude markers and boosters. For example:

(1) Not surprisingly, I was able to demonstrate *that membership in cohesive subgroups was linked to teachers' background characteristics and their sentiments and behaviors (E-SOC-SN-01)*.

To sum up, there is no use of *I* in Chinese and no use of *the author* in English, suggesting a more explicit expression of authorial stance in English.

### 4.2. The use of stance markers in the corpora across disciplines

#### 4.2.1. The quantitative presentation of stance markers

Now, let us turn to the disciplinary variable (see Table 4). Distinctions of the use of stance makers in soft science RAs and hard science RAs were found in the Chinese sub-corpus (χ^2^ = 26.410, df = 3, *p* < 0.05) and English sub-corpus (χ^2^ = 39.763, df = 3, *p* < 0.05). Soft science RAs employed more stance markers than hard science RAs both in the Chinese sub-corpus (67.64 > 32.36%) and English sub-corpus (69.4 > 30.4%). Thus, more than two-thirds of the stance markers in our corpus occurred in soft science RAs. It was also revealed that similarities in the use of stance markers in different disciplines were shared in the Chinese sub-corpus and English sub-corpus. Hedges occurred more frequently in soft science RAs (12.65 > 10.32% in the Chinese sub-corpus and 25.77 > 22.61% in the English sub-corpus). Moreover, self-mentions were much more favored by soft sciences writers (18.83 > 2.58% in the Chinese sub-corpus and 32.57 > 20.58% in the English sub-corpus). On the other hand, hard science RAs had a stronger intention to use boosters (37.42 > 32.41% in the Chinese sub-corpus and 34.38 > 23.99% in the English sub-corpus) and attitude markers (49.68 > 36.11% in the Chinese sub-corpus and 22.43 > 17.67% in the English sub-corpus).

#### 4.2.2. The qualitative analysis of the functions of stance markers

Hedges are necessary for both Chinese and English soft science writers to etch the interpretative feature of the claims. The writers exploited hedges to show a tentative position and withhold the commitment as opinions instead of assertive statements to weigh the degree of precision and open discursive space for readers. The most frequently exploited hedging device in total was modal verbs (69% of the total in soft sciences) such as *might, could*, and *would*. For example:

*(12) On the whole, the Florence lecture* would seem to be *a model of a successful intercultural lecture given by a culturally sensitive person (E-AL-ESP-09)*.
*(13) [The labor relation* might *present a diversified development tendency] (C-SOC-YS-13)*.

On the other hand, boosters are indispensable in Chinese and English hard science RAs to help stress the shared background information, show the confidence and certainty of the claims, and also emphasize the novelty and significance of the study. Words and phrases such as *can, prove*, and *in fact* allow writers to achieve their writing purposes. For instance:

(14) In fact*, an overlapping of binary decisions* can *create 21 (or with tolerated errors: 24) classes (E-BIO-BS-14)*.
*(15) [The simplification of this model was* proved to be *tenable through the thermal balance test] (C-ME-JMEE-03)*.

Additionally, Chinese and English writers in hard sciences had a tendency to use attitude markers. The main functions of attitude markers are to correspond the results with research questions or previous studies and to show the importance or limitation of the study both in soft and hard sciences (see Example 16). In addition, they are used to appeal to the attention of real practitioners such as teachers, researchers, and policymakers in soft sciences (see Example 17).

*(16) [The results of CFD analysis provide an* important *theoretical basis for improving the efficiency of centrifugal pump and expanding its operating range] (C-ME-FL-8)*.
*(17) Thus*, it is no surprise *that more and more* policymakers *in the medical and public health fields have suggested social inclusion as a potential direction of change (E-AL-AROS-15)*.

In view of self-mentions, there is a remarkably higher frequency in soft sciences than in hard sciences. Chinese and English writers in soft sciences preferred to use *I* to construct an appropriate persona and to exert the *inclusive we* to involve the readers and show proximity to the disciplinary community (see Examples 18 and 19). Undoubtedly, the *exclusive we* was used both in soft and hard sciences to show the authorial presence and present the findings (see Example 20).

*(18) In sum*, I present *place as evolving, idiosyncratic, and dynamic, and best approached holistically (E-SOC-AJOR-15)*.
*(19) [*We *are moving toward[sic] the twenty-first century, which many people say is the century of Asia and the Pacific] (C-SOC-YS-04)*.
*(20) In this paper*, we introduced *a duality-based two-level a posteriori error estimate in a general framework for non-linear time-dependent PDEs (E-ME-CMAME-15)*.

It is demonstrated that the stance markers in the RA conclusions display resemblances and variations across cross-linguistic/cultural and cross-disciplinary perspectives. This merits our further consideration, which will be discussed in the next section.

## 5. Discussion

Successful academic writing cannot be accomplished in one go but is impacted by several factors during the writing process. This sub-section discusses how both cultural and disciplinary factors play important roles in the use of stance markers in the conclusion sections of RAs. It will also be argued that some other factors, ranging from the individual to social factors, are influential in the choice of stance markers.

### 5.1. Cultural/linguistic factors affecting the use of stance markers

According to McCambridge (2019), cultural/linguistic variation should be taken into consideration in the studies of meta-discourse in academic writing. Thus, we will first analyze the culture/language difference. It is observed that English writers prefer to include more stance markers in their RAs than their Chinese counterparts overall. This result is consistent with previous English–Chinese comparative studies (e.g., Loi and Lim, 2013; Mu et al., 2015; Chen and Zhang, 2017) and the study of English in comparison with other languages/cultures (e.g., Vassileva, 2001; Vold, 2006; Mur-Dueñas, 2011), indicating that writers of international English RAs tend to exert more rhetorical devices to present their findings more stringently and persuasively by the balanced use of four sub-categories of stance markers to cater for the broad socio-cultural and heterogeneous readership. Another tentative explanation, as shown in Işik-Taş's (2018) study, maybe that English scholars appear to be more competent and familiar with rhetorical devices to “identify themselves with the discourse conventions of international journals” (p. 36) to deal with the competitiveness of publishing internationally, which contributes to more varieties and occurrences of stance markers. Thus, the occurrence of the four sub-categories in the English sub-corpus is more varied, which supports the claim that English writers have a better competence in balancing the stance markers to render the findings and claims in a more convincing and reader-oriented way (Crismore et al., 1993). Moreover, English writers make more linguistic choices to express their stance so that they can better level the weight of each sub-category. However, Chinese writers receive less instruction on how to negotiate their stance in RAs but receive more education on how to present the content, which is in line with Bulgarian writers in Vassileva's (2001) study. Furthermore, Chinese writers tend to avoid the use of strategies to show their stance explicitly, following the guidance of universities, which aligns with Işik-Taş (2018)' study that Turkish universities discouraged the explicit use of authorial identity strategies in their national journals. Therefore, Chinese writers, influenced by the high power distance culture of China (Hofstede, 2001), employ fewer stance strategies than English writers to adopt an impersonal presentation of knowledge to show relative objectivity and respect for authorities in the academic community. Another plausible explanation for the lesser use of stance strategies in Chinese articles might be the impact of language characteristics, namely, the fewer derivational and inflectional variations of Chinese characters compared to English words.

In terms of the specific use of stance markers, which concurs with previous studies (Hu and Cao, 2011; Yang, 2013; Mu et al., 2015; Chen and Zhang, 2017), English writers have a marked tendency to use hedges while Chinese writers are inclined to use boosters. In English RA writing, writers are influenced by Anglo-Saxon/American culture (Abdollahzadeh, 2011; Mur-Dueñas, 2011) in which clarity, correctness, and preciseness are underscored. Thus, English writers harness more hedges to make a cautious, precise, and appropriate commitment (Hu and Cao, 2011) and present the limitations of the study or the possibility of rejections so as to eschew responsibility and provide the findings more precisely for the matching of the real world (Abdollahzadeh, 2011). Additionally, logical and rational reasoning is a virtue in Western culture, which gives rise to the practice of writers “questioning one's own as well as others' ideas and beliefs” (Hu and Cao, 2011, p. 2,804). Thus, hedging, being an appropriate choice to leave space for alternative discussion or even disagreement, could be regarded as a tactful way to gain community acceptance and solidarity (Abdollahzadeh, 2011; Mu et al., 2015). On the contrary, influenced by the Chinese culture of high-context communication of *mianzi* and *hanxu* (Loi and Lim, 2013) and *zhongyong* (golden mean, which is considered to be the highest level of virtue by Confucius (551–479 BC) and Confucian scholars), Chinese writers deploy hedges dominantly to show humility, deference, modesty, and respect to avoid face-threatening acts and embarrassment (Yang, 2013), as opposed to English writers.

Second, boosters are recognized by both English and Chinese writers. However, unlike their use of hedges, Chinese writers employ notably more boosters than their English counterparts. As noted in previous studies (Hu and Cao, 2011; Chen and Zhang, 2017), Confucius and Taoism have a great impact on Chinese writers' academic writing, for “verbal debate and argumentation are not meaningful tools for understanding truth and reality” (cf. Hu and Cao, 2011, p. 2,804), leading to their lesser use of hedges for disputation and discussion but greater use of boosters to be assertive in their statements. In addition, in the high-context culture of China (Hofstede, 2001), facts and knowledge are based on prior or previous knowledge and authority (Chen and Zhang, 2017), thus making Chinese writers show certainty and confidence in their findings and assure the truth and reality of the knowledge by boosters. Finally, similar to Malay (Loi et al., 2016) and Spanish (Mur-Dueñas, 2011) writers, Chinese writers are more audacious in exercising boosters for the shared background in the local and homogeneous community.

Surprisingly, despite having little use in the whole RAs (Mu et al., 2015) and even no use in RA introductions (Loi and Lim, 2013), attitude markers have been favored by Chinese writers, and they appear as the most frequently used stance markers in our study. This difference could be attributed to the small size of the corpus and different sections of RAs. However, our result, consonant with Loi et al.'s (2016) study, shows that the aim of the conclusion section is to highlight the importance of the study (e.g., with 102 occurrences showing importance out of the total occurrences of 194). In addition, influenced by the Chinese culture of shared community that shows respect for authority and high power distance, Chinese writers exert attitude markers to show their agreement with previous studies and appeal for the obligation for further study as a shared academic community.

For self-mentions, English writers deploy a greater use of *I* to exhibit their responsibility, authority, and personal commitment (Kuo, 1999; Hyland, 2001), which is congruent with the findings of previous research (Walková, 2019; Chen, 2020). This may be ascribed to the influence of the Aristotelian claims of directness or explicitness (Abdollahzadeh, 2011). More importantly, in a writer-responsible culture (Dahl, 2004; McCambridge, 2019), English writers choose a direct and explicit way to show their authorial stance by the use of *I*. Another plausible reason may lie in the effect of individualism (Crismore et al., 1993; McCambridge, 2019), suggesting that the writers are aware of whom they are and what they want to do by catering to the presumed readers. Conversely, Chinese writers use more *inclusive we* to show modesty and collective identity and to hide the individual, which shows the key notion of collectivism in Chinese culture (McCambridge, 2019; Chen, 2020). Furthermore, Chinese culture is reader-responsible (Loi and Lim, 2013). The use of *we* can tone down ownership, mitigate responsibility, and make the statements seemingly objective (Mu et al., 2015). Particularly, the use of *we* in single-author RAs and the use of *the author* depicts the writers' distance from the authorial persona and reduces the role of writer-self in the text, which is in line with previous studies (Mu et al., 2015; Chen, 2020).

### 5.2. Disciplinary factors affecting the use of stance markers

Dahl (2004) proposes that “academic writing reflects national as well as disciplinary culture” (p. 1,807), which may give an account of the discipline difference affecting the use of stance markers. The result that in the soft sciences, both in the Chinese sub-corpus and English sub-corpus, more stance markers are utilized in RA conclusions than in the hard sciences overall is not surprising when disciplinary norms and conventions are taken into consideration. This suggests that soft sciences tend to be subjective, interpersonal, interpretative, and explanatory, while hard sciences are inclined to be objective, impersonal, informative, and routinized (Hyland, 2001; Jiang, 2017; Zou and Hyland, 2020). Therefore, it might be elaborated that soft science writers receive more literary training, and thus they have more choices to negotiate themselves in academic writing. However, hard science writers emphasize the training of experimental or methodological processes so that they have no strong awareness to present their stance in academic writing. Furthermore, this could be ascribed to the size of the two sets of data, as suggested by Loi and Lim (2013), that soft sciences articles are nearly twice the length of hard sciences, allowing writers more space to take their stance.

As for the four sub-categories of stance markers, the difference between soft and hard sciences is similar to the one between the Chinese and English sub-corpus. More hedges and self-mentions are employed in soft science RAs both in the Chinese sub-corpus and English sub-corpus. This might be explained by the fact that more discursive practices are applied based on writers' personal expertise and experience in soft sciences (Zou and Hyland, 2020). The construction of knowledge in soft sciences relies more on the writers' personal observation and explanation to recast knowledge as tenable and tentative as possible. Therefore, soft science writers have to show their findings and opinions prudently and precisely and try harder to establish an agreement with readers. To achieve this aim, hedges are frequently used in soft science RA conclusions to seek agreement and safety and leave room for discussion (Hyland, 2005; Abdollahzadeh, 2011). In addition, the feature of intertextuality (Charles, 2003; Jiang, 2017) in soft sciences is an indication of seeking agreement and solidarity with readers or searching for consensus (Warchał, 2010) to make their statements more persuasive. This also accentuates the use of hedges in soft sciences. On the contrary, the use of stance markers should craft the writers' persona (Dong and Buckingham, 2018) or as a projection of the authors' proximity (Jiang, 2017) in their academic writing that aligns with disciplinary culture and convention. In soft sciences, as demonstrated by Hyland (2005), the readership may not be as cohesive as in hard sciences when the knowledge is reconstructed in a new context. This provides the opportunity for more overt and explicit expression and evaluation to show the contribution and significance of the study and personal credibility in jotting down more convincing academic writing. Accordingly, the use of first-person pronouns plays a paramount role in successful academic writing (Hyland, 2001). The frequent use of *I* in soft sciences reflects the writers' purpose to gain credit, highlight contributions, and present an explicit stance (Hyland, 2005). The use of *we* can construct a suitable persona by producing proximity to the readership and disciplinary community, for *we* can involve either writers and readers or disciplines as a whole (Kuo, 1999; Hyland, 2001; Jiang, 2017) and stamps the writer's presence onto their arguments and engages the readers (Hyland and Jiang, 2019). Generally, the more explicit use of self-mentions in soft sciences is in agreement with previous studies (Charles, 2003; Jiang, 2017).

Meanwhile, the use of stance markers in hard sciences is distinct from that in soft sciences, for each science has its own fundamental principles and norms regarding the construction and attainment of knowledge (Zou and Hyland, 2020). Hard science writers in the Chinese sub-corpus and English sub-corpus take their stance through a high frequency of boosters and attitude markers in their RAs. This could be manifested by the fact that hard sciences are based on a shared understanding of a phenomenon, and thus readers, familiar with prior research, accept the proposition as a fact instead of reducing the certainty or confidence to hedge claims (Hyland, 2005). Likewise, hard science RA findings, which are based upon strict procedures, methods, and empirical demonstration from lab observation, are emphasized by a strong claim (Zou and Hyland, 2020). However, different from Dong and Buckingham (2018), attitude markers are very frequently employed in our study for the reason that novelty and significance should be highlighted either by showing importance or agreement with former studies to make the stance clearer and more persuasive (Charles, 2003). In addition, the communicative purposes of texts determine the discursive practices (Bhatia, 2004). Therefore, the conclusion section, one of whose communicative purposes is to recommend further study or provide guidance for practical operation, deploys attitude markers to achieve this purpose. For instance, as indicated in Yoon and Römer's (2020) study, writers in hard sciences choose to guide readers to interpret findings by attitude markers more explicitly than the ones in soft sciences. Finally, hard science writers exercise more *we* rather than *I* to keep objectivity and show they are irrespective of responsibility (Hyland, 2001). It should be noted, however, that the use of *we* offers evidence of an authorial stance by crafting an argument that seems to be objective but in fact provides emphasis (Liardét and Black, 2019). This indicates writers' awareness that writing is an interaction (Kuo, 1999) to negotiate social relations (Hyland, 2005). Thus, writers achieve the community identity construction and meanwhile keep a balance between presenting personal claims and disciplinary knowledge and practices (Charles, 2006).

### 5.3. Other factors affecting the use of stance markers

As pointed out by Yakhontova (2006) and underlined by Ädel (2022), the difference between the rhetorical strategies employed by different groups of writers can be explained not only by cultural and disciplinary factors but also by varied factors, from the individual experience, personality, preference, and ideology to broad institutional, social, and dynamic developmental factors.

Hyland (2005) maintains that a writer's individual personality, preference, and confidence would impinge on the use of stance markers. As suggested in our study, the single case of *I* in hard sciences shows the writer's preference for statement claiming. Also, the sequential use of *yes* (using *yes* three times to emphasize the results) in one RA demonstrates the writer's preference and confidence in making their claim. Individual experience in academic writing apparently plays a role in stance-taking in RAs. As illustrated by previous studies (e.g., Crismore et al., 1993; Liardét and Black, 2019), expert writers are inclined to deploy more stance markers in their academic writing, while inexperienced writers dominantly maintain an impersonal approach by using few stance markers. This is substantiated by the occurrence of stance markers in two RA conclusions, with 40 occurrences in one experienced writer's conclusion but only two occurrences in another less experienced writer's conclusion.

Another factor is the requirement of institutions, either the publishing house or the writer's own institution. Bhatia (2004) and Jiang (2017) propose that academic writing should be influenced by institutions to publish or be subservient to the rules. For example, there is no use of self-mention markers in our corpus in the *Energy Conversion and Management* journal and *Chinese Journal of Ecology*. Also, the length of words and characters is regulated in each journal, with a total of 15,312 words being the most in the *American Journal of Sociology* and 2,762 characters being the least in the *Journal of Mechanical and Electrical Engineering*. Writers are also placed under stress by their own institutions to publish in international journals, as noted by Loi and Lim (2013). Thus, writers from different backgrounds have to improve their competence to negotiate their stance and keep the information objective as much as possible.

Also, writers have to accept acculturation in academic writing (Chen, 2020) from the English language. English, as the international language and lingua franca for academic writing (Loi and Lim, 2013), has a bearing on Chinese academic writing conventions (Chen and Zhang, 2017). Thus, Chinese writers need to embrace pertinent writing awareness by taking English academic writing rules into consideration. Meanwhile, with the improvement in teaching, training, and learning, writers are more mature in a particular domain of research so that they have more choices to express their stance (Hyland and Jiang, 2016). As a result, there is an increase in the use of boosters and varieties of stance markers but a decrease in the use of hedges in RA writing, which could also be explained by the fact that academic conventions are also dynamic (Bhatia, 2004; Chen and Zhang, 2017). Thus, writers have to change their strategies and expressions of stance-taking. Furthermore, with the broadening of the academic group, the competition in publishing and maintaining a career is increasing (Sheldon, 2019; Chen, 2020), and the promotional aspect is more salient (Hyland and Jiang, 2019), which puts the burden on writers to express themselves more explicitly through stance markers in academic writing.

Finally, academic writing is rooted in social reality. Hu and Cao (2011) postulate that more use of boosters is associated with the development of Chinese technology. With a clearer and more regulated process of operating experiments due to the new technological instruments, writers would have more confidence in expressing their stance. This could also be reflected in applied linguistics, which involves more empirically grounded and quantitative studies (Hyland and Jiang, 2016). Indeed, China has put more emphasis on technology ever since COVID-19 and proposed the goal of *aiming for the frontiers of sciences and technology*. This could be an influential factor affecting the use of stance markers by Chinese writers in the process of the construction of academic writing.

## 6. Conclusion

In this article, we have investigated the employment of stance markers in English and Chinese soft and hard science RAs. It was found that RAs in the two corpora used four categories of stance markers to present the results, highlighting the significance and expressing authorial persona, which are the salient generic features of RA conclusions. The considerable usage of stance markers in English and soft science RAs reveals the cultural and disciplinary conventions for academic writing.

This study has implications for EAP teaching and EAL/EFL/ESL writing. Our findings indicate that the divergent use of stance markers may not only arise from the cultural and disciplinary factors, as highlighted by previous scholars (e.g., Mu et al., 2015; Chen and Zhang, 2017; Alghazo et al., 2021; Ädel, 2022), but also by the individual, institutional, social, and dynamic developmental ones. Such findings can be drawn upon by EAP and EAL/EFL/ESL teachers and practitioners to foster students' proper academic writing awareness and caution that academic writing, although static and fixed to some extent, is a dynamic form of social interaction (Jiang, 2017) through which writers project their stance, construct disciplinary knowledge, consolidate solidarity with putative readers, and reflect their cultural or disciplinary identity. Thus, academic writing teachers and non-native and novice student writers should bear in mind the diversified rhetorical features of stance markers along with the analysis of underlying reasons among the variations.

In addition, as the last chance to recast the significance and show the limitations to guide future studies, the conclusion section should make a balance between the use of different categories of stance markers and the display of objective information and interpersonal negotiation for knowledge construction and community acceptance (Abdollahzadeh, 2011). Thus, it is important for non-native and novice writers to write an informative and succinct conclusion section, on the one hand, to maintain their own authorial stance, on the other.

Admittedly, the current study has some limitations in terms of the size of the corpora and the objects for comparison. It is suggested that future research use a large-size three corpora comparison, namely, L1 English, L2 English by Chinese writers, and L1 Chinese, to explore the overall features of stance markers throughout research articles. Additionally, as the construction of a stance is complex, future studies should include the triangulation of writers' interview data to perform further analysis.

## Data availability statement

The raw data supporting the conclusions of this article will be made available by the authors, without undue reservation.

## Author contributions

LD and PH conceived the initial idea, designed the study, worked out the initial manuscript and revised the subsequent versions, and proofread the manuscript. PH collected the data, analyzed the data, drafted the manuscript, and finalized the manuscript for submission as the corresponding author. All authors have read and agreed to the published version of the manuscript.

## Footnotes

^1^To clarify, all the Chinese examples extracted from the sample texts were translated by the authors.
