# Supplementary material for: “We may conclude that:” a corpus-based study of stance-taking in conclusion sections of RAs across cultures and disciplines
Source: Front Psychol. 2023 May 5;14:1175144. doi: 10.3389/fpsyg.2023.1175144 (PMC10196383; doi:10.3389/fpsyg.2023.1175144)
Supplement: Supplementary file 1 [file Data_Sheet_1.docx]

## **Appendix A: Source journals**

**English journals**

*Applied Linguistics*

English for Specific Purposes

Journal of Second Language Writing

Second Language Research

*Sociology*

American Journal of Sociology

Annual Review of Sociology

Social Networks

*Mechanical Engineering*

Computer Methods in Applied Mechanics and Engineering

Energy Conversion and Management

Mechanics of Materials

*Biology*

Biologicals

Biosystems

Journal of Theoretical Biology

**Chinese journals**

*Applied Linguistics*

Foreign Language Teaching and Research

Foreign Language World

Modern Foreign Languages

*Sociology*

Youth Studies

Population and Development

Chinese Journal of Population Science

*Mechanical Engineering*

Journal of Mechanical and Electrical Engineering

Fluid Machinery

China Mechanical Engineering

*Biology*

Acta Ecologica Sinica

Chinese Journal of Ecology

Plant Physiology Journal

## **Appendix B: Stance markers in corpora**

**Stance markers in the English sub-corpus**

**Hedges:** *some, partly, little, may, would, could, might, seem, possible, possibly, possibility, likely, perhaps, probable, probability, assume, posit*

**Boosters:** *demonstrate, prove, determine, do + verb, define, claim, clear, clearly, certain, certainly, in fact, able, most, indeed, no doubt, needless, evident, obvious, yes, of course, notable, can, will, must*

**Attitude-markers:** *important, useful, note-worthy, valuable, remarkable, contribution, promising, beneficial, play a role, especially, in particular, particularly, good, optimal, interesting, suitable, appropriate, fortunately, unfortunately, accurate, success, successful, surprise, surprising, efficient, need, necessary, should, hope, call for, remain, have/has to, recommend*

**Self-mentions:** *I, we, our, my ,us, me*

**Stance markers in the Chinese sub-corpus**

**Hedges:** *一定程度(yidingchengdu)，一些(yixie)，大约(dayue)，比较(bijiao)，似乎(sihu)，是否（shifou），能否(nengfou)*

**Boosters:** *证明(zhengming)，取决于(qujueyu)，决定(jueding)，解决(jiejue)，填补(tianbu)，强调(qiangdiao)，确定(queding)，必然(biran)，事实上(shishishang)，关键(guanjian)，确实(queshi)，无疑(wuyi)，明显(mingxian)，可以(keyi)，能(neng)，必将(bijiang)，肯定是(kendingshi)，必须(bixu)*

**Attitude-markers:** *重要的(zhongyaode)，有利于(youliyu)，有益于(youyiyu)，有意义的(youyiyide)，有价值的(youjiazhide)，显著(xianzhu)，启示(qishi)，尤其(youqi)，特别(tebie)，较好(jiaohao)，突破(tupo)，首次提出(shoucitichu)，大幅(dafu)，需要(xuyao)，应该(yinggai)，希望(xiwang)，期待(qidai)，必要(biyao)，值得(zhide)，不容忽视(buronghushi)*

**Self-mentions:** *我们(women)，笔者(bizhe)*
